# Co-occurrence pattern and function prediction of bacterial community in Karst cave

**DOI:** 10.1186/s12866-020-01806-7

**Published:** 2020-05-29

**Authors:** Yiyi Dong, Jie Gao, Qingshan Wu, Yilang Ai, Yu Huang, Wenzhang Wei, Shiyu Sun, Qingbei Weng

**Affiliations:** 1 School of Life Sciences, Guizhou Normal University, Guiyang, 550001 Guizhou China; 2 CAS Key Laboratory of Tropical Forest Ecology, Xishuangbanna Tropical Botanical Garden, Chinese Academy of Sciences, Mengla, 666303 Yunnan China; 3 University of Chinese Academy of Sciences, Beijing, 100049 China; 4 Center of Conservation Biology, Core Botanical Gardens, Chinese Academy of Sciences, Mengla, 666303 Yunnan China; 5 Department of Bioengineering and Biotechnology, Huaqiao University, Xiamen, 361021 Fujian China

**Keywords:** Bacterial community, Co-occurrence network, Function prediction, Karst, Zhijin cave, Oligotrophy, Tourism, 16S rRNA gene

## Abstract

**Background:**

Karst caves are considered as extreme environments with nutrition deficiency, darkness, and oxygen deprivation, and they are also the sources of biodiversity and metabolic pathways. Microorganisms are usually involved in the formation and maintenance of the cave system through various metabolic activities, and are indicators of changes environment influenced by human. Zhijin cave is a typical Karst cave and attracts tourists in China. However, the bacterial diversity and composition of the Karst cave are still unclear. The present study aims to reveal the bacterial diversity and composition in the cave and the potential impact of tourism activities, and better understand the roles and co-occurrence pattern of the bacterial community in the extreme cave habitats.

**Results:**

The bacterial community consisted of the major Proteobacteria, Actinobacteria, and Firmicutes, with Proteobacteria being the predominant phylum in the rock, soil, and stalactite samples. Compositions and specialized bacterial phyla of the bacterial communities were different among different sample types. The highest diversity index was found in the rock samples with a Shannon index of 4.71. Overall, Zhijin cave has relatively lower diversity than that in natural caves. The prediction of function showed that various enzymes, including ribulose-bisphosphate carboxylase, 4-hydroxybutyryl-CoA dehydratase, nitrogenase NifH, and Nitrite reductase, involved in carbon and nitrogen cycles were detected in Zhijin cave. Additionally, the modularity indices of all co-occurrence network were greater than 0.40 and the species interactions were complex across different sample types. Co-occurring positive interactions in the bacteria groups in different phyla were also observed.

**Conclusion:**

These results uncovered that the oligotrophic Zhijin cave maintains the bacterial communities with the diverse metabolic pathways, interdependent and cooperative co-existence patterns. Moreover, as a hotspot for tourism, the composition and diversity of bacterial community are influenced by tourism activities. These afford new insights for further exploring the adaptation of bacteria to extreme environments and the conservation of cave ecosystem.

## Background

The adaptation of life to extreme environments, which are steady or fluctuating habitats and not conducive for human, has recently become a hot topic for research. Extremophiles can colonize the extreme environment, and they are the sources of novel biomolecules and metabolic pathways [1, 2]. Karst caves are the terrain formed by the process of soluble rock dissolution [3]. Meantime, it is one of the extreme habitats with harsh conditions, for instance, nutrient-limitation, darkness, and high humidity [4]. Microorganisms can also act as the primary producers and sustain the cycles of substance and energy by chemoautotrophic and photosynthetic activities, which are normally found in various cave habitats [4–7]. These microorganisms participate in the geochemical cycles through inorganic chemical reactions in the cave ecosystems [8, 9]. However, our understanding of the microbial diversity, distribution patterns, and the roles of microorganisms in Karst caves is incomplete [3]. Exploring the microbial diversity and composition of Karst cave is particularly important for understanding the ecosystem and biodiversity in such extreme environment.

Previous studies about microorganisms in caves mostly focused on the microbial community composition, including describing and comparing the number of taxa, relative abundances, and alpha diversity [10], or investigating the microbial communities from different sample types such as wall surface [11], sediments [12], water [13–15], rock, and air [15]. However, the co-occurrence patterns of complex microbial communities are primarily unclear [16]. The co-occurrence network analysis allows us to explore the interactions between coexisting taxa in complex and diverse microbial communities [10, 17, 18]. Recently, it has been used to analyze microbial communities in complex habitats from gut intestine [19] to cave [15, 16] and ocean [20]. Network analysis approaches provide new insights for the interaction networks, structure, and niches distribution of communities in the Karst caves [10, 17, 21].

Zhijin cave exhibits the complex evolutionary process and the pattern of the plateau Karst in Guizhou province since the Paleogene. Meanwhile, it is a typical developmental zone and the microcosm of the plateau Karst in China [22]. Due to a large number of rare stalactites and the fantastic Karst landscape, Zhijin cave has become a tourist cave as well since 1983 and a global geological park. With the presence of tourists, the cave ecosystems such as the CO_2_ concentration, temperature, composition of microbial communities have affected by human activities [23–26]. However, the changes of microbial communities resulted in the loss of pigment on the surface of the walls and sediments in caves [27–29]. Hence, exploring the composition of bacterial community is important to conservation of cave. Recently, it was verified that the environment conditions in Zhijin cave have been changed, including the upward trend of the CO_2_ concentration and air temperature [30, 31], decreased relative humidity [31]. Moreover, the culturable bacterial composition in water has been impacted by tourism activities in Zhijin cave [32]. However, so far, the bacterial diversity and roles in the Zhijin cave are still unclear. To better understand the function and co-occurrence pattern of the bacterial community in the oligotrophic Karst cave, further reveal the potential impacts of tourism activities on bacterial diversity and composition in cave habitats, in this study, 16S rRNA high-throughput sequencing technology was used to analyze multiple different sample types, which would better reflect the composition of bacteria communities in Zhijin cave ecosystem. The bacterial composition, network structure, and function of the bacterial communities were further compared. We focus on the following objectives: (i) the composition characteristics of bacterial communities in the Karst tourism cave, (ii) their potential functions and metabolism pathways across different sample types, and (iii) the co-occurrence network patterns of bacterial communities in Zhijin cave.

## Results

### Sequence data

A total of 900,491 reads for 16S ribosomal RNA sequencing were successfully obtained from all the samples collected from seven sites in Zhijin cave (Supplemental file 1: Table S1). After removing low-quality, replicate, and potential chimera tags, we obtained 827,376 tags ranging from 39,403 to 93,528 per sample site. Based on 97% similarity, 27,421 OTUs were obtained across all samples.

### Bacterial community composition

**Fig. 1:**
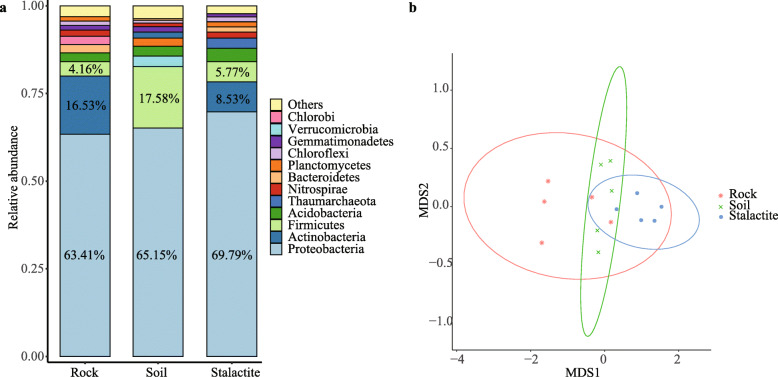
Bacterial community composition. **a** Relative abundances of the 10 most abundant phyla in each sample. The relative abundance not shown in chart if fewer than 4%. **b** Non-metric multidimensional scaling (NMDS) of bacterial community in three sample types

A total of 54 phyla, 750 genera, and 407 species were determined in three sample types. We selected the top 10 most abundant phyla in each sample type to further measure the composition of bacterial community. Their relative abundances were shown in Fig. 1a. We found that 9 phyla (Proteobacteria, Actinobacteria, Firmicutes, Acidobacteria, Nitrospirae, Bacteroidetes, Planctomycetes, Chloroflexi, and Gemmatimonadetes) were shared by all sample types. Among the top 10 predominant phyla in samples, Chlorobi was not detected in soil samples; Verrucomicrobia was not found in rock samples; Thaumarchaeota was only obtained in stalactite samples. The Proteobacteria had the highest relative abundance in the bacterial community across all sample types.

The relative abundances of Proteobacteria were 63.41, 65.15, and 69.79% in rock, soil, and stalactite samples, respectively. In both rock and stalactite samples, Actinobacteria remained to be the second abundant phylum in the bacterial communities (16.53 and 8.53%, respectively), and then followed the phylum Firmicutes (4.16 and 5.77%, respectively). Whereas, in soil samples, the second abundant phylum in bacterial community was Firmicutes (17.58%), which was more abundant than that in rock and stalactite samples. The Abundances of other phyla were less than 4% across the three sample types.

At the species level, the NMDS ordination method showed that bacterial communities were separately different among the three sample types (Fig. 1b). The results were consistent with the significant test that the means of the distances were considered as different (*P* < 0.01) among the three sample types. Besides, the results from ANOSIM showed that the differences of bacterial community compositions were significant between rock and soil samples (R = 0.70, *P* < 0.01), rock and stalactite samples (R = 0.74, *P* < 0.01), as well as stalactite and soil samples (R = 0.79, *P* < 0.01).

### Bacterial groups with statistical differences

**Fig. 2:**
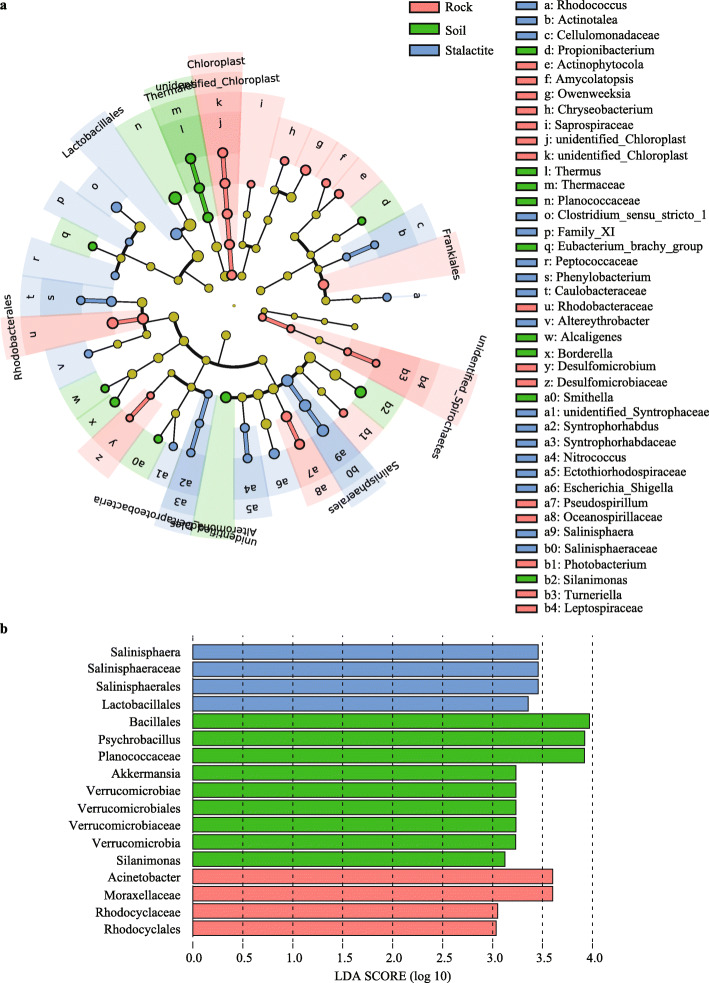
The results of LEfSe analysis. **a** Cladograms indicating the phylogenetic distribution of bacterial lineages associated with the samples. **b** Indicator bacterial group significantly differentiated across the three sample types with LDA values higher than 3

The LEfSe analysis was performed for comparing bacterial communities to find the specialized bacterial groups within each type of the samples. The cladogram (Fig. 2a) showed that 2 phyla, 2 classes, 8 orders, 15 families, and 26 genera were significantly variable across the three sample types. From phylum to species, there were 15, 9, and 17 groups of bacteria enriched in rock, soil, and stalactite samples, respectively. Indicator groups represented the abundance differentiation of the bacterial group (LDA value of 3 or higher) among the three sample types (Fig. 2b). There were 9 differentially abundant bacterial groups in soil samples (e.g. Bacillales, *Psychrobacillus*, and Planococcaceae). A total of 4 bacterial groups (e.g. Acinetobacter, Moraxellaceae, and Rhodocyclaceae) were significantly more abundant in rock samples, and 4 taxa (e.g. Salinisphaeraceae and Lactobacillales) were overrepresented in stalactite samples.

### Venn diagram and bacterial diversity

To further obtain insight into the differences of bacterial communities across the three sample types, the Venn analysis of the OTUs was performed, which demonstrated that OTUs differed across the three sample types (Supplemental file 2: Figure S1). The number of site-specific OTUs ranged from 175 (soil samples) to 435 (rock samples), and a total of 322 OTUs were shared among all three sample types. According to the OTUs identified at different levels of taxon, the Simpson’s index, Shannon’s index, and Simpson evenness were calculated. The Simpson’s index (0.87–0.91) and Shannon’s index (3.63–4.71) indicated that the level of diversity varied among three sample types. The average Simpson indices were 0.91, 0.89, and 0.87 in rock, soil, and stalactite samples, respectively. The average Shannon’s indices were 4.71, 3.63, and 4.17 in rock, soil, and stalactite samples, respectively. The greatest bacterial diversity was observed in rock samples. However, the LSD test showed that the diversity indices were not significantly different across the three sample types.

### Inferred bacterial function by PICRUSt

**Fig. 3:**
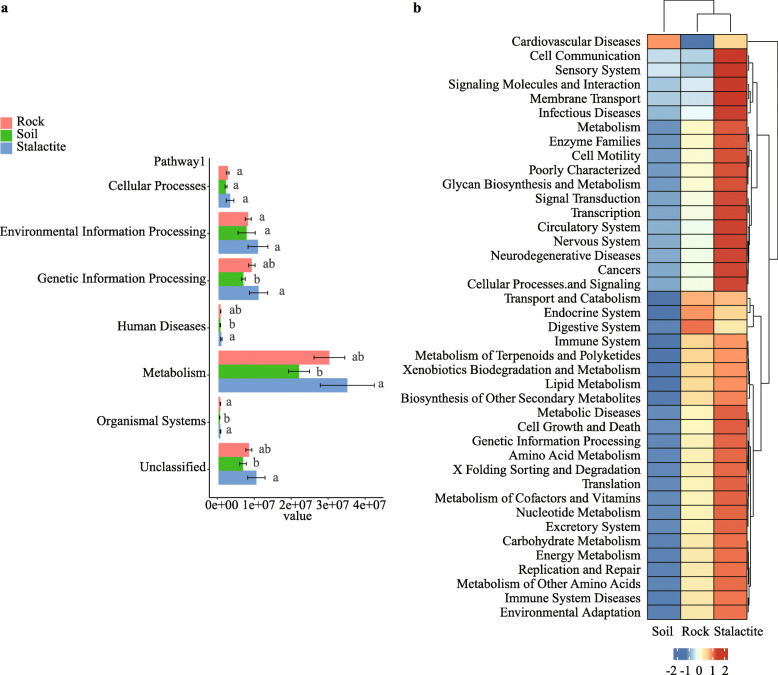
The PICRUSt predicted function in samples. **a** Predicted function of bacteria among the three sample types. **b** The second level of KEGG pathway was shown in the heatmap

Based on the PICRUSt analysis, the results of KEGG (Kyoto Encyclopedia of Genes and Genomes) pathway abundance in each sample were obtained (Fig. 3a). A total of 6 functional modules that represented approximately 86% of the entire dataset in samples were detected, including cellular processes (4%), environmental information processing (15%), genetic information processing (15%), human diseases (1%), metabolism (49%), and organismal systems (1%). The most abundant functional module was metabolism across the three sample types. In generally, the functional modules were richer in stalactite samples than those in other sample types.

A total of 41 pathways were predicted across the three sample types (Fig. 3b). Among them, there were 37 pathways in stalactite samples (e.g. cell communication, sensory system, signaling molecules and interaction, and membrane transport), which were more abundant than those in the other two sample types. Moreover, cellular processes and organismal systems (e.g. transport and catabolism, endocrine system, and digestive system) were overrepresented in rock samples. In soil samples, only the cardiovascular diseases pathway was more abundant than that in other sample types.

In addition, to understand the potential metabolism of carbon and nitrogen reactions in which the bacteria participated in, we also aimed to detect the relative encoding genes and enzymes for carbon and nitrogen metabolisms in the samples of Zhijin cave. It showed that carbon metabolism pathways, including glycolysis pathway, pentose phosphate pathway, methanogenesis pathway, and 6 carbon fixation pathways (reductive pentose phosphate cycle, 3-hydroxypropionate bicycle, reductive citrate cycle, hydroxypropionate-hydroxybutyrate cycle, reductive acetyl-CoA pathway, and dicarboxylate-hydroxybutyrate cycle), possibly existed in Zhijin cave. The predicted relative enzymes were shown in Supplemental file 3: Table S2, e.g. hexokinase [EC:2.7.1.1], glyceraldehyde 3-phosphate dehydrogenase [EC:1.2.1.12], and pyruvate kinase [EC:2.7.1.40] involved in the glycolysis pathway; hexose-6-phosphate dehydrogenase [EC:1.1.1.47 3.1.1.31], glucose-6-phosphate isomerase [EC:5.3.1.9], and transaldolase/glucose-6-phosphate isomerase [EC:2.2.1.2 5.3.1.9] involved in the pentose phosphate pathway; heterodisulfide reductase subunit D [EC:1.8.98.1], methyl-coenzyme M reductase alpha subunit [EC:2.8.4.1], and F420-non-reducing hydrogenase large subunit [EC:1.12.99.- 1.8.98.5] involved in the methanogenesis pathway; and enzymes related to carbon fixation pathways (e.g. 4-hydroxybutyryl-CoA dehydratase [EC:4.2.1.120 5.3.3.3], ribulose-bisphosphate carboxylase [EC:4.1.1.39], and malonyl-CoA reductase [EC:1.2.1.75 1.1.1.298]).

Moreover, total of 5 related nitrogen metabolism pathways, including nitrogen fixation process, denitrification pathway, dissimilatory nitrate reduction pathway, assimilatory nitrate reduction reaction, and complete nitrification pathway, were revealed. The predicted related enzymes were shown in Supplemental file 4: Table S3, e.g. nitrogenase delta subunit [EC:1.18.6.1], nitrogenase iron protein NifH [EC:1.18.6.1], and ammonia monooxygenase subunit C [EC:1.13.12.-] involved in the nitrogen fixation process; hydroxylamine dehydrogenase [EC:1.7.2.6] involved in the nitrification pathway; hydroxylamine oxidase [EC:1.7.3.4], nitrite reductase (NO-forming) [EC:1.7.2.1], and nitrate reductase (cytochrome) [EC:1.9.6.1] involved in the denitrification pathway; nitrate reductase (cytochrome) [EC:1.9.6.1] and nitrate reductase/nitrite oxidoreductase beta subunit [EC:1.7.5.1 1.7.99.-] involved in the dissimilatory nitrate reduction pathway; ferredoxin-nitrate reductase [EC:1.7.7.2] and assimilatory nitrate reductase electron transfer subunit [EC:1.7.99.-] involved in the assimilatory nitrate reduction reaction.

### Bacterial co-occurrence network analysis

**Fig. 4:**
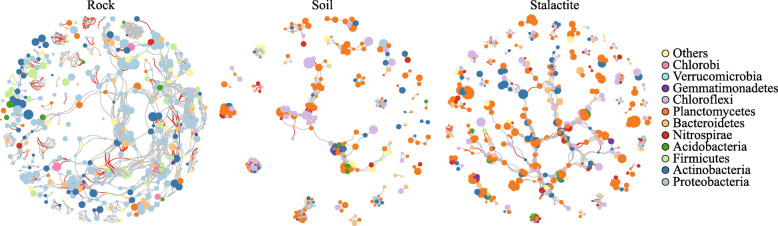
The co-occurring network analysis of the bacterial communities across the three sample types. The nodes are colored by phylum level, the size of each node is proportional to the relative abundance of specific genus level. The color of each edge is positive and negative of correlation coefficient, grey represents positive correlation, and red represents negative correlation. The thickness of each edge is proportional to the correlation coefficient (Spearman’s *r* > ±0.8 and *P* < 0.01)

The correlation coefficient (*r* > ±0.8, *P* < 0.01) co-occurrence network analysis (Fig. 4 & Supplemental file 1: Table S4) showed that the edges in the network included 1127 strong positive correlations and 184 negative correlations in the rock samples; 1353 strong positive correlations and 32 negative correlations in the soil samples; and 1263 strong positive correlations and 145 negative correlations in the stalactite samples. The modularity indices of the three sample types were all greater than 0.40, which suggested that the co-occurrence networks of bacterial communities had a strong modular structure and complex species interaction across the three sample types in Zhijin cave [33]. Comparing to a randomized network, three non-random co-occurrence networks were observed in Zhijin cave (*P* < 0.01).

In the co-occurrence network, the rock samples presented a shorter average path length (the shortest among all possible pairs of nodes) and a lower diameter (the longest of the shortest paths among all pairs of nodes) (1.98 and 2.00) than those in soil samples (3.66 and 16.00) and stalactite samples (7.84 and 23.00), which reflected a more efficient information processing and substance transmission among the species in rock samples. Furthermore, the C score (co-occurrence index) was 0.33, and the robustness (stability of community) of bacterial community was 0.86 in the rock samples. The greater values of co-occurrence index indicated that bacteria were highly exclusive, and the large robustness represented that the bacterial community was more stable. Compared with the rock and stalactite samples, the clustering coefficient (the proportion of neighboring nodes that can be reached through the nodes connecting other neighbors) was greater in the soil samples (0.97). The bacterial network had lower values of co-occurrence index (0.24) and robustness (0.77) in the soil samples. These results reflected that a relatively high rate of cooperation was formed by a higher clustering coefficient and more bacteria were co-occurred in the community with a low stability in soil samples.

The node degree (the number of ties with other nodes) reflects the role of node. According to the node degree of each node, the keystone taxa in each sample type were captured. The largest node degree was found with *Salinarimonas* (466 links), *Cloacibacterium*, and *Marinospirillum* (both 17 links) in rock samples; *Pasteurella*, *Dietzia*, *Hungatella*, and *Beggiatoa* (all 37 links) in soil samples; and *Streptomyces*, *Pseudolabrys*, and *Ignavibacterium* (all 20 links) in stalactite samples, suggesting that these groups were more prominent than the other groups with fewer links in the same network.

## Discussion

### Composition of bacterial community in different sample types

The environment of Zhijin cave was occupied by diverse bacteria. Though the Karst cave is an extreme habitat with nutrition deficiency, light limitation, and oxygen deprivation [3, 4], our results showed that 54 phyla with 750 genera were detected from the rock, stalactite, and soil samples, indicating a rich bacterial diversity in the cave. The Proteobacteria was the predominant group across all the three sample types. Proteobacteria, as a group of microbes responding to unstable carbon sources, was found to be the most abundant in some other cave ecosystems and various environments as well [25, 34–37]. The Firmicutes was the second abundant phylum found in soil samples in Zhijin cave, possibly due to their resistance towards nutrient stress and capability of survival under most extreme habitats [25, 38]. For other bacteria groups found in samples, the Actinobacteria is considered as the production source of bioactive compounds, but knowledge of their diversities in caves is very limited [4]. The Bacteroidetes and Acidobacteria are capable of decomposing organic compounds in environments [39, 40], and the Acidobacteria, specifically, as oligotrophic organism was negatively correlated with nutrient levels [41, 42]. As autotrophic green non-sulfur bacteria, the Chloroflexi, which can fix CO_2_, was a prevalent phylum and frequently found in caves [43, 44]. The presence of the Nitrospirae is crucial for the nitrogen cycle, since several nitrite oxidizers are present in Nitrospirae, carrying out nitrification and supplying nitrogen for oligotrophic environments [45]. In addition, the Thaumarchaeota, containing ammonia-oxidizing archaea [46], could obtain energy by oxidizing ammonia and fixing carbon in oligotrophic environments [47, 48].

Cave habitat is the important factor affecting bacteria survival, as a result of which, different bacterial communities were found in various niches in cave environments [49]. The observed divergent compositional structure and diversity of bacterial communities in different extreme cave environments might be due to the differences in cave environments, sampling mediums, and analysis methods, to a certain extent [50]. The indicator bacteria were significantly different among three sample types. The soil samples had the most abundant indicator taxa (e.g. Bacillales, *Psychrobacillus*, and Planococcaceae), and the LEfSe analysis also revealed more specific bacterial groups in soil samples than those in the other sample types. These indicator taxa in samples implied that these bacterial groups specifically functioned in their special habitats. For example, Bacillales is the common group in soil [51] and on mineral surface [52], and some Bacillales [53, 54] and *Psychrobacillus* [55] are able to degrade oils in contaminated soil. *Salinisphaera* was the indicator taxon in stalactite samples, and previous studies illustrated that several strains of *Salinisphaera* are halophilic bacteria [56, 57] and capable of fixing CO_2_ by using organic carbon sources [58]. It implied that *Salinisphaera* might be involved in the process of stalactite formation by fixing carbon cycle. Further, Moraxellaceae strains have the ability to bear the natural transformation and most of them usually inherently reside on the mucosal membranes in humans and other animals [59].

It was worth noting that there are some hints that the bacteria diversity and composition in Zhijin cave might be influenced by the changed ecosystem because of human activities. Firstly, we observed the relatively lower bacterial diversity indices in rock samples [11, 15], soil samples [45, 60], or stalactite samples [11] from Zhijin cave than those in many natural caves currently reported. Several studies using culture-based method also uncovered that microbial diversity in show caves was lower than that in natural caves [25, 26]. In some instances, the diversity of fungi was influenced by the level of anthropogenic disturbance, in which lower diversity was found in areas with heavy disturbance and higher diversity was found in sites with moderate disturbance [26]. It hinted that the lower bacterial diversity might be the consequence of tourism in Zhijin cave. Secondly, the predominance of Proteobacteria in Zhijin cave was consistent to the previous reports in tourist cave ecosystems [25, 34–36]. The Firmicutes dominated in bacterial community in natural cave or areas with less tourism influence, and conversely [25]. Lastly, several microbial groups of anthropogenic origin [61], such as *Lactobacillus*, *Bacteroides*, *Staphylococcus*, and *Moraxella* genera, also observed in our samples (data not shown). Here, we further inferred that the impact of human activities on cave ecosystem. With the human access and mass activities of tourists, certain energy and exogenous bacteria are potentially transported into the cave system following the dropping skin, hair, sweat, and clothing [25, 27, 49, 50]. The organics introduced from human activities provide new nutrients and habitats for some bacteria, moreover, resulted in the changes of bacterial community in cave. In additionally, the human activities affected the cave microenvironment and led to increased CO_2_ concentration, raised temperature, and decreased relative humidity [12, 26]. These further have a profound influence on the processes of the cave forming and the bacterial composition in cave. Integrating the tourism activities, the changed the environment conditions in the Zhijin cave, such as concentration CO_2_ [30, 31], and these traits of bacterial diversity and composition, which further confirmed that the cave ecosystem were disturbed by human activities in Zhijin cave. As indicators of cave tourism, monitoring the bacterial diversity and composition is crucial to comprehend human impacts and the changes of bacteria in cave habitats [62, 63].

### Prediction of bacterial functions using PICRUSt

To respond to the severely limited resource, chemolithotrophic microorganisms create biogenic energy and nutrients in the cave ecosystem using sulfur and metal irons [64, 65]. Besides, other metabolic processes such as nitrogen fixation, carbon fixation, and carbon mineralization allow microorganisms to maintain the cave ecosystem [63, 66]. Similarly, we found that the bacteria can survive through various metabolic processes in Zhijin cave. The co-existent bacteria could involve in a variety of complex metabolic reactions in the cave habitat, which was supported by the relative genes and enzymes for carbon and nitrogen metabolism pathways detected by PICRUSt analysis across the three sample types.

We obtained 6 functional modules in the samples, and the predominant module was the metabolism. However, due to the lack of photosynthesis in cave environments, the autotrophic bacteria may act as both the primary producer and the common energy input [67–69]. Carbon fixation is considered as a watershed between the heterotroph and autotroph organisms. Autotrophic organisms can fix CO_2_ through Calvin cycle, which are widely distributed in environments [70]. The ribulose-bisphosphate carboxylase, which is the rate-limited enzyme in Calvin cycle, was observed in the samples, suggesting that Calvin cycle appeared to exist in the environment of Zhijin cave. Moreover, bacteria may utilize several other specific metabolic pathways in extreme environments. The 4-hydroxybutyryl-CoA dehydratase is the indicator enzyme of 3-hydroxypropionate/4-hydroxybutyrate cycle [47, 71] and dicarboxylate cycle/4-hydroxybutyrate cycle [72], involving CO_2_ fixation in archaea [47] and Thaumarchaeota [73]. According to the previously reported study, 4-hydroxybutyryl-CoA dehydratase was shown abundant in oligotrophic environments and contributed to the processes of carbon assimilation in cave environments [74]. The 3-hydroxypropionate bicycle is another autotrophic carbon fixation pathway, as a new CO_2_ fixation pathway in *Chloroflexus* [44, 45]. The malonyl-CoA reductase is the key enzyme of 3-hydroxypropionate bicycle, which was observed in several green non-sulfur bacteria for autotrophic CO_2_ fixation [44].

For the nitrogen cycle, previous studies mostly focused on the role of bacteria in soil, rock, aquatic, and other oligotrophic conditions [75]. Similarly, we also detected the relative enzymes for nitrogen cycle from samples in Zhijin cave. These enzymes involved nitrogen fixation, denitrification, nitrate reduction, nitrate reduction, and nitrification were predicted. Due to the limitation of nitrogen resource in the cave ecosystems, bacteria could survive by specific strategies and metabolic pathways [34, 76–78]. Some autotrophic bacteria in Nitrospirae, Chloroflexi, and Chlorobi identified in Zhijin cave, might transfer the N element into the nitrogen cycle by the nitrogenase NifH or chemoautotrophic process, utilize inorganic compounds such as ammonia by the nitrification, and promote the nitrogen cycle in environment [6]. In addition, the nitrite reductase is a key enzyme in the dissimilatory denitrification [79], the bacteria catalyze the reduction of nitrite to nitric oxide in the environments by using the nitrite reductase, and may contribute to reduce nitrogen loss in oligotrophic cave [32]. The ammonia-oxidizing bacteria containing hydroxylamine oxidase probably come from the Nitrospirae and Thaumarchaeota found in Zhijin cave, are the predominant ammonia oxidizers and participate in the ammonia oxidation process which is the first key step of nitrification [5, 32, 80]. In a conclusion, the presences of these metabolic enzymes suggested that bacteria, including some autotrophic bacteria, can survive by participating in specific metabolic pathways in the nutrient-limited Zhijin cave.

In addition, the abundances of metabolic pathways were divergent across the three sample types in Zhijin cave. This probably indicated that the bacterial metabolic activities are divergent, functional bacteria involved in different dynamic activities could drastically shift in different samples and habitats in the cave system, and specific microorganisms play a key role in energy transformation and different geological cycles [81].

### Co-occurrence pattern of bacterial communities

The bacterial co-occurrence networks had different topological properties and complex species interactions across all the three sample types in Zhijin cave in our study. We observed that a great number of edges, high robustness, and modularity in each sample, indicating that steady and complex interactions and strong modular structure were present in the bacterial communities [33]. Moreover, the low values of average path length and diameter in rock samples illustrated that the information and the substances were quickly transmitted among species in the bacteria communities. This implied that the bacteria had a higher transmission powers in the rock samples than in the other samples [82]. Whereas, the highest co-occurrence index detected in the rock samples implied a lower co-existence degree in the bacterial communities. For the soil and stalactite samples, the clustering coefficients were 0.97 and 0.74 in the bacterial networks respectively, suggesting the relatively strong correlation of co-occurrence network [16]. The lower co-occurrence index, higher clustering coefficient, and lower negative edges all hinted that the bacterial community in the soil samples possessed higher cooperation. Previous study reported that most ecological networks had a low value of connectance [83], the same results of which were obtained in all the samples from Zhijin cave as well in this study. As a matter of fact, networks with low connectance indicate a power-law distribution [83, 84].

Though the keystone taxa were divergent in different sample types, the bacterial groups had a number of links with the other groups in the same communities. It implied that these bacteria taxa perform key roles or are responsible for the interactions among the communities through specific metabolic activities. For example, *Salinarimonas rosea* sp. nov. is a halotolerant bacterium and capable of reducing the nitrate [85]; *Pasteurella* could oxidize organic compounds and assimilate sulfur compounds [86]; and *Streptomyces* may produce various and complex secondary metabolites [87]. These keystone taxa involve in complex metabolic cycles and supply primary or secondary metabolic products for cooperators of the communities in cave.

Furthermore, the networks were consisted of a number of positive edges but fewer negative edges in each sample type in Zhijin cave. This species co-occurrence patterns were displayed by the co-occurring and positive interactions among different phyla. Several previous reports uncovered that metabolic exchanges were detected in nutrients-limited environments [88–90]. These examples indicated that the metabolic cooperation could drive the co-occurrence pattern of bacteria and shape the compositions of communities [88]. Thereby, the different co-occurrence network structures of the bacterial communities could be explained by the interdependencies in the microorganisms from the collected samples. The consistent result was revealed by the network descriptors with disparate values of networks and the compositions of the communities in Zhijin cave.

## Conclusions

Network analysis allows us to explore the composition and interaction of a community. In this study, our results confirmed that the tourism activities could influence the bacterial diversity and composition in Zhijin cave. In the dark and oligotrophic cave, the bacteria could co-exist through positive interactions and cooperation by participating in diverse metabolic pathways. This study develops a better understanding of the adaptation and interaction patterns of bacterial communities in extreme habitats, and provides the evidence for the development and conservation of cave system.

## Methods

### Study sites and sample collection

**Fig. 5:**
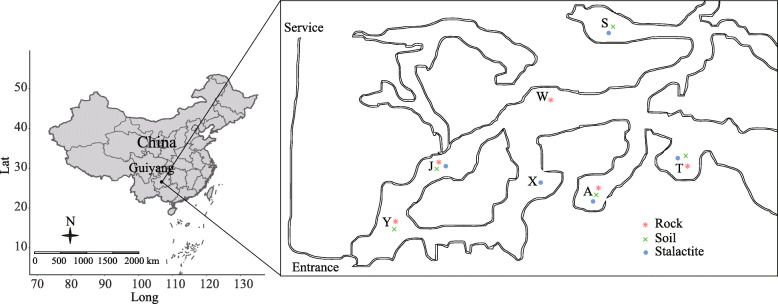
Distribution of sampling sites. A, J, S, T, W, X, Y represent different sampling sites

Zhijin cave is located in Guizhou Province in China (26°38′31″-26°52′35″N, 105°44′42″-106°11′38″E). The average annual temperature is 18–20 °C, the average annual humidity is 90%, and the average CO_2_ concentration is 0.20%. Three types of samples, including rock, soil, and stalactite, were collected in December 2016. Each type of sample was collected from five different sites (Fig. 5). To collect the samples on the rocks and stalactites, forty-five swabs moistened with sterile deionized H_2_O were used to swab the surface (4 cm^2^ per swab) of the rocks or stalactites at individual sampling site [74]. Soil samples (200 g) were collected from the surface soil (0–10 cm) with a spade at each sampling site. The swabs and soils were placed in tubes, which were then capped, placed on ice, and immediately transported back to store at − 20 °C for further DNA extraction in the laboratory.

### DNA extraction and sequencing

Genomic DNA was extracted [12] from 30 swabs or 1 g soil. The sample material was resuspended in 1500 μL of TES buffer (0.3 M sucrose, 25 mM Tris-HCl pH 8.0, 0.25 mM EDTA pH 8.0) supplemented with 50 μL Lysozyme (20 mg/mL). The mixture was vortexed for one minute and incubated for 1 h at 37 °C. Then 30 μL Proteinase K (20 mg/mL) and 200 μL 10% sodium dodecyl sulphate were added to the tubes, followed by vortex for 3 min and incubation for 2 h at 55 °C. Subsequently, 200 μL 5 M sodium chloride and 400 μL CTAB/NaCl were added, and the mixture was vortexed for 5 min and incubated for 30 min at 65 °C. The resulting lysate was extracted with phenol: chloroform: isoamyl alcohol (volume 25:24:1). The DNA was precipitated by adding 0.1 volume of 3 M Na-acetate (pH 4.8) and 0.6 volume of isopropanol. The DNA pellets were air-dried under − 20 °C temperature for 2 h, resuspended in 100 μL ddH_2_O, and stored at − 20 °C.

Finally, amplicon sequencing was conducted with the extracted DNA using an Illumina MiSeq platform following the protocols described by previous study [74]. The gene-specific primers 515F (5′-GTGCCAGCMGCCGCGGTAA-3′) and 806R (5′-GGACTACHVGGGTWTCTAAT-3′) were developed based on the V4 region of the bacterial 16S rRNA gene [﻿91﻿]. DNA amplification was carried out by polymerase chain reaction (PCR) with the Thermo Scientific Phusion High-Fidelity PCR Master Mix (New England Biolabs, UK), and the extracted DNA solution was diluted to 1 ng/μL for amplification with the specific primers. The amplification was carried out in a 30 μL reaction volume consisting of 15 μL of 2 × Phusion Master Mix (New England Biolabs, UK), 10 μL DNA template, 3 μL of each primer (2 μM), and 2 μL molecular water. Reaction was performed with 1 min at 98 °C, 30 cycles of 10 s at 98 °C, 30 s at 50 °C, 30 s at 72 °C, and a final extension at 72 °C for 5 min. Amplicons from three reactions for each sample were excised from gels, pooled, and purified using GeneJET Gel Extraction Kit (Thermo Scientific). The sequencing was performed on the Illumina MiSeq platform at the Novogene Bioinformatics Technology (Beijing, China).

### Data processing, assembly, and annotation

Bacterial raw reads were produced by the Illumina MiSeq platform, the raw sequences were assembled for each sample, and low-quality sequences were filtered using QIIME [92]. The OTUs (Operational Taxonomic Units) table for each sample were clustered at the 97% similarity following the Uparse (http://drive5.com/uparse/), and the OTUs were classified and annotated based on the clustering results using the RDP database (http://rdp.cme.msu.edu) offering aligned and annotated for bacterial 16S rRNA sequences [93].

### Statistical analysis

The relative abundances of the top 10 most abundant phyla in each sample were analyzed. To compare the similarity of composition in bacterial community, a NMDS (non-metric multidimensional scaling) analysis and a test of significance among sample types of bacterial community were performed. Variations of species composition among samples were tested by ANOSIM (An Analysis of Similarities) at species level and calculated using 999 permutations in *vegan* package. To assess the indicator bacterial group specialized in three sample types, LEfSe (Linear discriminant analysis effect size) analysis was performed in python 2.7 environment. Shared and unique OTUs among the three sample types were used to generate a Venn diagram in *VennDiagram* package. Statistical analysis on α diversity index by OTUs richness was performed with *vegan* package in R [94], the diversity indices of different sample types were compared with ANOVA (one-way analysis of variance), and the mean of diversity indices was tested by the LSD (Least Significant Difference) test in *agricolae* package [95]. The functional profiles of the bacteria were obtained by PICRUSt (Phylogenetic Investigation of Communities by Reconstruction of Unobserved States) analysis [96], based on the results from the normalize_by_copy_number.py analysis, and then the taxonomic file was uploaded to perform the functional prediction online (http://huttenhower.sph.harvard.edu/galaxy/). A co-occurrence network analysis at genus level was performed to explore the linkage of the different bacterial community in *igraph* package [97]. We calculated the spearman correlation matrix and filtered the correlation coefficient, which were both lower than ±0.8 and not at the significant level. Then the co-occurrence network was plotted. In order to describe the structure of the network, the average path length, diameter, clustering coefficient, module, co-occurrence index (C score), robustness, and counting up the node degrees were measured in *igraph* and *bipartite* packages [98]. Besides, the networks in collected samples and null model (r2dtable) were compared to test the network distribution.

## Supplementary information

**Additional file 1: Table S1.** Sample list and sequencing information. **Table S4.** Co-occurrence network descriptors for bacterial communities across the three sample types.

**Additional file 2: Figure S1.** Venn diagram showing the exclusive and overlap of bacterial OTUs across the three sample types.

**Additional file 3: Table S2.** List of the encoding genes and enzymes involved in carbon metabolism.

**Additional file 4: Table S3.** List of the encoding genes and enzymes involved in nitrogen metabolism.

## Data Availability

All datasets are presented in the main text and the additional file. Besides, the raw datasets and R code are available on Github digital repository (https://github.com/dongyiyi/Data-from-Co-occurrence-pattern-and-function-prediction-of-bacterial-community-in-Karst-cave). All raw data can be acquired from the corresponding author on request.

## Abbreviations

PICRUSt: Phylogenetic Investigation of Communities by Reconstruction of Unobserved States
EDTA: Ethylene Diamine Tetraacetic Acid
CTAB: Cetyltrimethylammonium bromide
KEGG: Kyoto Encyclopedia of Genes and Genomes
NMDS: Non-metric Multidimensional Scaling
LEfSe: Linear Discriminant Analysis Effect Size
LSD: Least Significant Difference
OTU: Operational Taxonomic Units
rRNA: Ribosomal RNA
PCR: Polymerase Chain Reaction
ANOVA: One-way Analysis of Variance
ANOSIM: An Analysis of Similarities
