# Supplementary material for: Co-occurrence pattern and function prediction of bacterial community in Karst cave
Source: BMC Microbiol. 2020 May 29;20:137. doi: 10.1186/s12866-020-01806-7 (PMC7257168; doi:10.1186/s12866-020-01806-7)
Supplement: Supplementary file 1 — Additional file 1: Table S1. Sample list and sequencing information. Table S4. Co-occurrence network descriptors for bacterial communities across the three sample types. [file 12866_2020_1806_MOESM1_ESM.docx]

Table S1 Sample list and sequencing information

| Samples | Raw reads | Total tags | Taxon Tags | Unique Tags | OTU number |
| --- | --- | --- | --- | --- | --- |
| Rock.A | 47262 | 44533 | 42586 | 1947 | 1928 |
| Rock.J | 54662 | 51793 | 49617 | 2176 | 1903 |
| Rock.T | 55281 | 51742 | 48223 | 3519 | 2296 |
| Rock.W | 58373 | 55535 | 53399 | 2136 | 1955 |
| Rock.Y | 54670 | 51953 | 47614 | 4339 | 2316 |
| Soil.S | 49789 | 46655 | 45760 | 892 | 694 |
| Soil.J | 46556 | 41784 | 41098 | 686 | 552 |
| Soil.Y | 50227 | 43173 | 32906 | 10267 | 1374 |
| Soil.T | 42647 | 40045 | 39373 | 669 | 609 |
| Soil.A | 42897 | 39403 | 36512 | 2891 | 1018 |
| Stalactite.T | 102582 | 65059 | 58664 | 6395 | 2541 |
| Stalactite.A | 72635 | 78156 | 75374 | 2782 | 2269 |
| Stalactite.J | 82231 | 74492 | 65499 | 8993 | 2657 |
| Stalactite.S | 86037 | 93528 | 86721 | 6807 | 3009 |
| Stalactite.X | 54642 | 49525 | 45448 | 4077 | 2300 |

A, J, S, T, W, X, Y represent different sampling sites in Zhijin cave.

Table S4. Co-occurrence network descriptors for bacterial communities across the three sample types.

| Network descriptors | Rock | Soil | Stalactite |
| --- | --- | --- | --- |
| Number of edges | 1311 | 1385 | 1408 |
| Average path length | 1.98 | 3.66 | 7.84 |
| Clustering coefficient | 0.08 | 0.97 | 0.74 |
| Diameter | 2.00 | 16.00 | 23.00 |
| Modularity index | 0.66 | 0.71 | 0.87 |
| Connectance | 0.02 | 0.04 | 0.01 |
| C score | 0.33 | 0.24 | 0.31 |
| Robustness | 0.86 | 0.77 | 0.85 |
