# Supplementary figures and images for: Co-occurrence pattern and function prediction of bacterial community in Karst cave
Source: BMC Microbiol. 2020 May 29;20:137. doi: 10.1186/s12866-020-01806-7 (PMC7257168; doi:10.1186/s12866-020-01806-7)

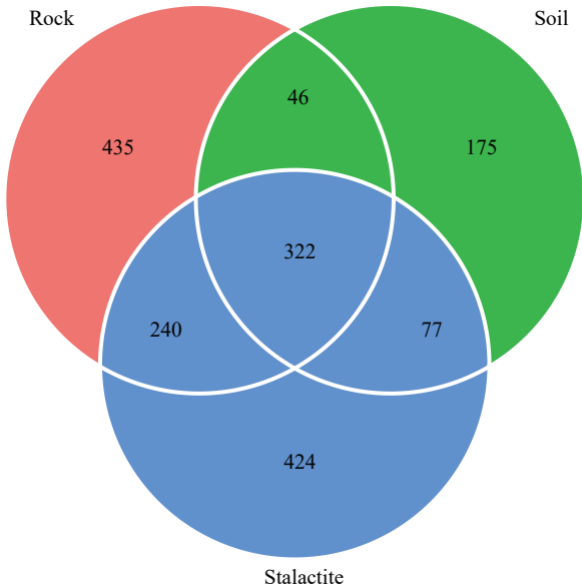

Supplement: Supplementary file 2 — Additional file 2: Figure S1. Venn diagram showing the exclusive and overlap of bacterial OTUs across the three sample types. [file 12866_2020_1806_MOESM2_ESM.pdf]
